# Gaps in transitional care to adulthood for patients with cerebral palsy: a systematic review

**DOI:** 10.1007/s00381-023-06080-2

**Published:** 2023-08-08

**Authors:** Devon L. Mitchell, Nathan A. Shlobin, Emily Winterhalter, Sandi K Lam, Jeffrey S Raskin

**Affiliations:** Division of Pediatric Neurosurgery, Department of Neurological Surgery, Lurie Children’s Hospital, Northwestern University Feinberg School of Medicine, 225 E Chicago Ave, Box 28, Chicago, IL 60611 USA

**Keywords:** Cerebral palsy, Transitional care, Transition, Pediatric, Adulthood, Complex care

## Abstract

**Purpose:**

The transition from pediatric to adult care can be complex and difficult to navigate for adolescents with cerebral palsy (CP). We aimed to assess the current state of transitional care for young persons with CP and delineate guidelines for best practice with opportunities for intervention.

**Methods:**

A systematic review was conducted using PRISMA guidelines to search PubMed, Embase, and Scopus databases. Articles were screened for relevance via title and abstract prior to full-text review.

**Results:**

Of 3151 resultant articles, 27 observational studies were included. Fourteen (52%) studies assessed clinical outcomes of patients with CP during and post-transition. Transition-associated poor outcomes included housing instability, unemployment, difficulty forming relationships, increased hospital admission rates, and decreased use of rehabilitation services. Factors associated with improved outcomes included family participation, promotion of self-efficacy, and meeting the adult team before transition. Nine (33%) studies conducted interviews with transition-age persons with CP. Key themes were a lack of transition preparedness, difficulty navigating the adult system, gaps in seamless care, and limited accessibility to specialists and environments suitable for patients with complex care needs. Four (15%) studies examined features of current transition services. Perceived barriers included poor communication within health service teams, limited adult providers accepting CP patients, and the lack of financial resources for specialized care. There was no standardized transition tool or approach.

**Conclusion:**

These findings underscore the importance of a planned transition process in optimizing long-term medical and psychosocial outcomes for persons with CP. Further research, including translational, team-based, and community-engaged research, are needed.

**Supplementary Information:**

The online version contains supplementary material available at 10.1007/s00381-023-06080-2.

## Introduction

Cerebral palsy (CP) is the most common childhood cause of mobility disability, with a prevalence of 2 to 2.5 per 1000 live births [1, 2]. Medical advancements have resulted in 40–90% of children with CP reaching adulthood, depending on disease severity, at which point care models historically limited to childhood may become inadequate [1, 3]. As a result, the number of young people with CP requiring transition to adult healthcare and developmental services has increased [4].

Young adults with CP require a broad period of transition from pediatric to adult care services. Additionally, transitional care is often complicated by a wide range of complex care needs, including neurodevelopmental and functional limitations [5–7]. Disease severity and associated impairments vary, requiring deeply personalized care throughout the individual’s lifespan [8, 9]. As a result, transition-associated goals must be curated to the patient’s capabilities and functional status. Unsuccessful transition to adult care is associated with the development of secondary disabilities [5], increased risk of treatment failure [10] or nonadherence [11], failure to establish care with adult providers [12], and increased rates of hospitalization [13, 14].

Despite the critical nature of effective transitional care and the serious negative health outcomes associated with inadequate transition, there is limited data characterizing the current state and development of transitional care paradigms for young persons with CP. A recent systematic review [15] examined gaps in transitional care interventions for young adults with childhood-onset neurologic disabilities but did not focus on disease-specific populations. Similarly, guidelines [16] for transitioning youth with complex care needs from pediatric to adult care have been established but are non-specific to young adults with CP. We conducted a systematic review to analyze the current state of transitional care for young persons with CP, including (1) healthcare-related, functional, and social outcomes of transition-aged patients; (2) patient, caregiver, and provider perspectives; and (3) structure and efficacy of existing transition-based interventions.

## Methods

### Search strategy

A systematic review was conducted using PRISMA (Preferred Reporting Items for Systematic Reviews and Meta-Analyses) guidelines [17] to investigate the current state of transitional care for young persons with CP and current healthcare attitudes. Three databases were searched in December 2020: PubMed MEDLINE (National Library of Medicine), Embase (Elsevier), and Scopus (Elsevier). Search strategies for Embase and Scopus were adapted from the PubMed search strategy shown in Supplementary Table 1. Date, study type, and language limits were not applied. Searches were conducted using controlled vocabulary (MeSH), keywords, and keyword synonyms.

### Screening

Duplicates were removed after search completion using the automated deduplication feature in Endnote X9 (Clarivate, London, UK). The remaining eligible studies were screened by title and abstract for relevance. Those selected for full-text review were screened using predetermined inclusion and exclusion criteria. Two authors, DLM and EW, completed both stages of screening. Conflicts were reconciled by discussion to achieve consensus.

### Inclusion and exclusion criteria

Inclusion criteria were publications in or translated into English, with full text and abstract available, studying a transition-age patient population (between 16 and 24 years old) with the diagnosis of cerebral palsy and assessing healthcare use, functional or social outcomes, perspectives on transitional care, or current implementations of transitional care practices. Studies reporting data on adult and pediatric patients or for cerebral palsy and additional pathologies were included if definite outcomes for transition-age patients with cerebral palsy could be identified. Exclusion criteria were as follows: abstracts only, secondary literature, not assessing transition-age patients with cerebral palsy, and not reporting on specified outcomes.

### Data extraction

Articles selected for final inclusion were assessed for bibliographic data, study design, participants, intervention where applicable, and outcome data. Outcome measures were specified a priori. The first primary outcome measure was transition readiness, which was assessed qualitatively via healthcare utilization metrics, social and functional outcomes, and patient, caregiver, and provider interviews and surveys. Healthcare utilization metrics included rates of primary care visits, hospital stays, specialist referrals and visits, and use of rehabilitation services. Social outcomes were assessed via level of social or community engagement, participation in support groups and activities, and relationship development. Functional outcomes were assessed via employment status, housing, and capacity to live independently, with consideration of Gross Motor Function Classification System (GMFCS) level. Key themes of interviews were summarized. Surveys ranged in structure and content, using various validated questionnaires, scales, or both. The second primary outcome measure was efficacy of existing transitional care interventions, which was assessed qualitatively via survey responses from program administrators, providers, patients, and caregivers.

### Statistical analysis

Data was analyzed descriptively. Means were calculated as weighted means and reported with range when available. As data was heterogenous by study design and execution, data were not pooled, and a meta-analysis was not conducted.

### Quality assessment

The income status for countries of study origin was determined using the World Bank designation [18]. Quality and risk of bias were assessed using the GRADE framework [19] and Cochrane ROBINS-I (Risk of Bias in Non-Randomized Studies of Interventions) tool [20] for each of the included studies. Overall risk of bias for this systematic review was inferred based on risk of bias of each included study.

## Results

### Search results

**Fig. 1:**
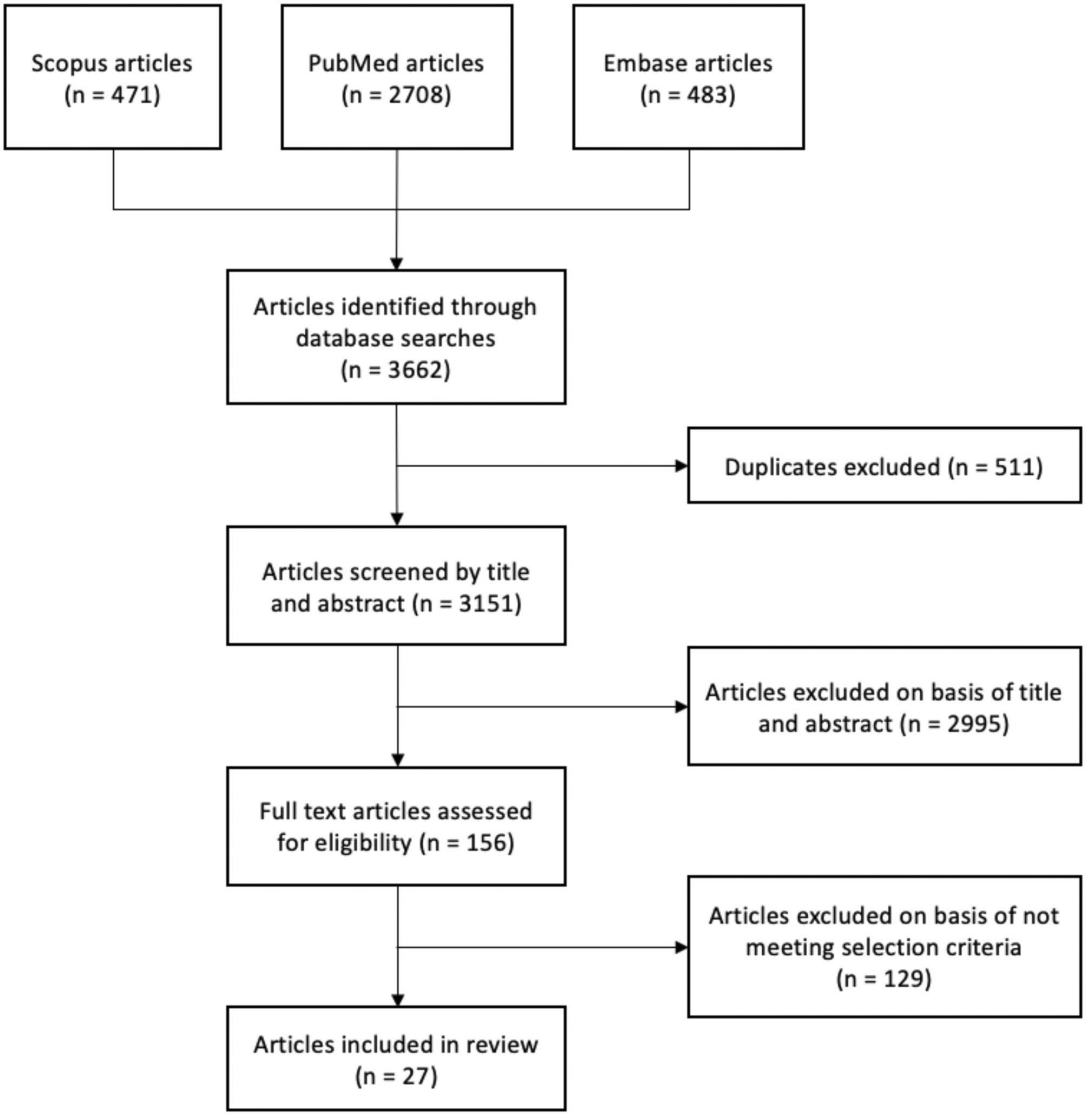
PRISMA flowchart outlining the search and review process used to identify and select articles for inclusion in the systematic review

The search identified 3151 articles, of which 27 were included (Fig. 1, Tables 1, 2, and 3) [21–47]. Study designs included longitudinal cohort (3, 11.1%), cross-sectional survey (10, 37.0%), qualitative interview (10, 37.0%), retrospective analysis (3, 11.1%), and retrospective cohort (1, 3.7%). All included studies originated from high-income countries, including the USA (9, 33.3%), the UK (7, 25.9%), Canada (4, 14.8%), Sweden (3, 11.1%), Scotland (1, 3.7%), Denmark (1, 3.7%), France (1, 3.7%), and the Netherlands (1, 3.7%). Twenty-three of the included studies [21–25, 27–29, 31–38, 40–44, 46, 47] (85.2%) assessed transition-aged persons with CP and four studies [26, 30, 39, 45] (14.8%) assessed transitional care interventions. The quality of most studies was moderate (12, 44.4%). Most studies had a moderate risk of bias (12, 44.4%), resulting in this review having a moderate risk of bias as well.

**Table 1:** Studies with transition-associated metrics as outcome measures included in the review

**Reference**	**Country**	**H/L/MIC**	**Study design**	**Quality**	**Bias risk**	**Patients, *N***	**Mean Age, yrs (range)**	**GMFCS level, *N***	**Cohort**	**Key findings**
Alriksson et al. (2014) [21]	Sweden	High	Longitudinal cohort	High	Low	102	20.6 median (18.3–23.7)	I, 38; II, 21; III, 13; IV, 10; V, 20	Participants of a CP follow-up program	Living arrangements differed significantly among GMFCS levels. 70% of participants with severe disabilities lived with their parents. 90% of employed participants had a GMFCS level of I–II
Colver et al. (2018) [29]	UK	High	Longitudinal cohort	High	Low	74	19.1 (16.1–22.0)	Not provided	Young persons with CP	Parent involvement was significantly associated with wellbeing. Satisfaction with services was significantly associated with promotion of health self-efficacy. Meeting the adult team before transfer was significantly associated with participation in arranging personal care
Donkervoort et al. (2009) [32]	Netherlands	High	Cross-sectional survey	High	Low	81	20.4 (18.0–22.0)	I, 63; II, 7; III, 5; IV, 5; V, 1	Young persons with CP with normal intelligence (IQ > 70)	Young adults with CP significantly lagged peers in development of housing, employment, and intimate relationships. 50% of participants did not visit a rehab physician in the previous year and only 33% visited a rehab physician in adult care
Goodman et al. (2011) [33]	USA	High	Cross-sectional survey	High	Low	1300	(18.0–21.0)	Not provided	Young persons with CP	Hospital utilization increased significantly among transitional age patients with CP in terms of number of annual discharges, inpatient days, and charges
Ko et al. (2004) [34]	UK	High	Qualitative interview	Moderate	Moderate	11	(15.0–17.0)	Not provided	Young persons with CP on school leave with physical disabilities	49 potential referrals to adult specialist services were identified, but 17 were not made as such services did not exist. Adult physiotherapy and OT services were under-provided
Liljenquist et al. (2018) [36]	USA	High	Retrospective analysis	Moderate	Moderate	35,290	(13.0–18.0 at wave 1, 21.0–26.0 at wave 5)	Not provided	Young persons with CP in school (wave 1) and out of school (wave 5)	59.4% of the youth utilized PT services; only 33.7% of them reported using PT since leaving secondary school. Female sex and use of a mobility device were significantly associated with PT use post-high school
McDowell et al. (2015) [37]	UK	High	Cross-sectional survey	Moderate	Moderate	123	16.2 (4.0–27.0)	IV, 55; V, 68	Young persons with CP and their parents	There was a significant decrease in access to specialists between the adolescent age group and the young adult age group
Merrick et al. (2015) [38]	UK	High	Cross-sectional survey	Moderate	Moderate	106	16.5 (14.0–18.9)	I, 25; II, 42; III, 16; IV, 11; V, 2; unclassified, 10	Young persons with CP	The median “gap” score between ideal and current care for physical environment and care processes was 1.0 when rated by young persons with CP. Parents’ satisfaction was significantly lower than their children’s
Blackman et al. (2013) [25]	USA	High	Cross-sectional survey	High	Low	80	(15.0–18.0)	Not provided	Parents of young persons with CP	29% reported that their doctors discussed their child eventually seeing adult providers. 42% reported their doctors have discussed changing healthcare needs as the child ages
Roquet et al. (2018) [41]	France	High	Retrospective analysis	Moderate	Moderate	512	(2.0–40.0 +)	I–III, 277; IV/V, 235	Family members and individuals with CP	Use of medication increased, while physical types of healthcare decreased with age, independent of GMFCS status
Solanke et al. (2018) [42]	UK	High	Cross-sectional survey	Moderate	Moderate	106	16.4 (14.0–18.9)	I, 53; II, 20; III, 15; IV/V, 18	Family members and individuals with CP	Highest areas of unmet needs were for management of pain, bone or joint problems, and speech, and were associated with increased severity of motor impairment and attending non-specialist education
Warschausky et al. (2017) [44]	USA	High	Cross-sectional survey	Moderate	Moderate	43	18.63	I, 19; II, 11; III, 4; IV, 9; V, 0	Parents and young persons with CP	TRAQ scores in the CP population indicated poor transition readiness for self-management but sufficient readiness in self-advocacy
Young et al. (2007) [46]	Canada	High	Retrospective cohort	High	Low	1064 total; youth 587, adults 477	Youth 15.4 (13.0–17.0); adults 26.3 (23.0–32.0)	Not specified for most of the sample	Youth and adults with CP	Adults had a significantly higher rate of GP visits and annual physicals compared to youth and a lower rate of specialist and pediatrician visits. Specialists provided 28.4% of youth visits but only 18.8% of adult visits
Young et al. (2010) [47]	Canada	High	Cross-sectional survey	High	Low	199 total; youth 129, adults 70	Youth 15.5 (13.0–17.0); adults 26.6 (23.0–33.0)	Youth: I–III, 68; IV/V, 61; adults: I–III, 39; IV/V, 31	Youth and adults with CP	SRH was reported to be excellent or very good by 57% of youth and 46% of adults

*N* refers to the number of patients in each study, *H/L/MIC* high/low/middle income country, *SRH* self-rated health, *CP* cerebral palsy, *GP* general practitioner, *TRAQ* Transition Readiness Assessment Questionnaire, *GMFCS* Gross Motor Function Classification System, *PT* physical therapy, *IQ* intelligence quotient, *OT* occupational therapy

**Table 2:** Qualitative interview or survey-type studies on patient, caregiver, or provider perceptions of their experiences with transitional care

**Reference**	**Country**	**H/L/MIC**	**Study design**	**Quality**	**Bias risk**	**Patients, *N***	**Mean age, yrs (range)**	**GMFCS**	**Cohort**	**Key themes**
Bagatell et al. (2016) [22]	USA	High	Qualitative interview	Moderate	Moderate	9	26.2 (19.0–33.0)	I, 4; II, 1; III, 0; IV, 2; V, 2	Young adults with CP who graduated from high school	Being thrust into adulthood, difficulty with navigating systems and services, understanding and managing my body, and dealing with stereotypes and prejudice
Bjorquist et al. (2015) [23]	Sweden	High	Qualitative interview	Low	High	12	(17.0–18.0)	Not provided	Young adults with CP	Looking forward to being an adult, but not feeling ready yet; belonging to a family means security but may be “too much”; socializing and love are necessary but not always possible; ADL are manageable but challenging; surrounded by support but don’t know what’s going on; hopes for the future but a desire for steppingstones
Bjorquist et al. (2016) [24]	Sweden	High	Qualitative interview	Low	High	10	(17.0–18.0)	I, 8; II, 3; III, 1; IV, 1; V, 2	Parents of young adults with CP	“Friction blisters chafing and healing during transition.” Five subthemes: concern and sorrow, stress and suffering in daily life, worries about what was to come, desire for help, strategies for coping and cohesion
Brandon et al. (2019) [27]	Canada	High	Qualitative interview	Moderate	Moderate	7	20.0 (18.0–30.0)	IV, 5; V, 2	Young adults with CP, parents of young adults with CP, pediatricians and PCPs	All participant groups reported transition challenges with respect to accessibility, the suitability of some primary care environments for caring for individuals with complex care needs, gaps in seamless care, and limited time and funding when receiving or providing primary care services to young adults with CP
Carroll et al. (2015) [28]	USA	High	Qualitative interview	Moderate	Moderate	9	(19.0–25.0)	Not provided	Young adults with CP	Expert novices, evidence/experience-based expectations, negotiating new systems, interdependence, and accepting less than was expected
Difazio et al. (2014) [31]	USA	High	Qualitative interview	Low	High	13	32.0 (24.0–44.0)	Not provided	Young adults with CP, parents of young adults with CP	Lost in Transition, Roadmap to Care, List of None, and One Stop Shopping
Lariviere et al. (2013) [35]	Canada	High	Qualitative interview	Low	High	14	20.9 (18.0–25.0)	Not provided	Young adults with CP	Transition envisaged with fear and apprehension, lack of cooperation between providers in the pediatric and adult healthcare systems, lack of support during transition, improper management of medical records, and feelings of abandonment
Normann et al. (2020) [40]	Denmark	High	Qualitative interview	Low	High	6	25.8 (21.0–31.0)	I, 2; II, 1; III, 1; IV, 2	Young adults with CP	Being a young adult, development in physical disability and new challenges in adulthood, and navigating the healthcare system
Stevenson et al. (1997) [43]	UK	High	Cross-sectional survey	Moderate	Moderate	74	Young (15.0–18.0), older (20.0–22.0)	Not provided	Young adults with CP, parents of young adults with CP	Carers expressed anxieties about the provision of services, and frustration in obtaining information about help

*N* refers to the number of patients in each study, *H/L/MIC* high/low/middle income country, *CP* cerebral palsy, *GMFCS* Gross Motor Function Classification System, *ADL* activities of daily living

**Table 3:** Studies assessing current implementations of transitional care or structured transition programs for young persons with cerebral palsy included in this review

**Reference**	**Country**	**H/L/MIC**	**Study design**	**Quality**	**Bias risk**	**Participants, *N* (type)**	**Key findings**
Bolger et al. (2016) [26]	USA	High	Cross-sectional survey	Moderate	Moderate	11 (clinics)	Top 3 perceived barriers to successful TOC were limited adult providers willing to accept CP patients, concern about the level of care in the adult healthcare system, and lack of financial resources. 55% of clinics had structured transition programs, but only one transitioned 100% of their patients to adult providers by age 22. 40% of clinics had transitioned < 25% of their patients with CP to adult providers by age 22. Only one clinic had an absolute upper age limit for seeing patients, and 36% of clinics accepted new patients older than 21. No respondents were “completely satisfied” with their transition process and only one was “moderately satisfied.”
Colver et al. (2018) [30]	UK	High	Longitudinal cohort	High	Low	85 (patients)	The nine proposed beneficial features of transition services were poorly provided. Fewer than half of services stated they provided an age-banded clinic, written transition plan, transition manager for clinical team, a protocol for promotion of health self-efficacy, or holistic life-skills training. Young people reported that they had not experienced the features which services said they provided. Agreement for written transition plan, holistic life-skills training and key worker was 30, 43, and 49%, respectively. Agreement was better for appropriate parent involvement, age-banded clinic, promotion of health self-efficacy, and coordinated team at 77, 77, 80, and 69% respectively
Morton et al. (2021) [39]	USA	High	Retrospective analysis	High	Low	1 (clinic)	More than 2/3 of families received services in seven categories: support primary care, specialty care, school, legal, community inclusion, healthcare financing, and providing medical care. Workplace, direct service providers and healthcare financing case workers received the least attention
Wright et al. (2015) [45]	Scotland	High	Qualitative interview	High	Low	13 (clinics)	Key areas in need of improvement were coordination and communication within health services and between health services and educational, social services and adult health services to which young people were transitioning

*N* refers to the number of participants in each study, *H/L/MIC* high/low/middle income country, *CP* cerebral palsy, *TOC* transition of care

### Patient demographics

The 23 included studies (85.2%) assessing transition-aged persons with CP consisted of 39,245 adolescent and young adult patients with cerebral palsy (Tables 1 and 2). The mean age of study participants at time of assessment ranged from 2 to 44 years old, with a weighted mean of 19.6 years. Nine studies [23–25, 28, 33, 34, 36, 41, 43] (39.1%) did not report a mean age. Six studies [32, 34, 37, 41, 43, 44] (26.1%) classified participants by cerebral palsy subtype, of which 72.7% (614/844) were spastic, 10.5% (89/844) were dyskinetic, 3.5% (29/844) were ataxic, 9.8% (83/844) were classified as “other,” and 3.5% (29/844) were unclassifiable. Additionally, 12 studies [21, 22, 24, 27, 32, 37, 38, 40–42, 44, 47] (52.2%) classified participants according to GMFCS level, of which 58.1% (757/1304) were levels I–III (mild/moderate impairment, ambulatory), 41.2% (537/1304) were level IV/V (severe impairment, non-ambulatory), and 0.8% (10/1304) were unclassifiable.

### Transition-associated healthcare metrics

Forty-two percent of parents of transition-age youth with CP reported their doctors having discussed the changing healthcare needs of their child as they aged [25]. However, only 28–29% reported that their doctors had discussed the eventual need to see adult providers or had provided help in coordinating transitional care [25]. Nonetheless, 64% of participants were able to formulate their care demands themselves [32]. Higher level of motor functioning, higher level of education, participation in activities, parent involvement, satisfaction with services, and meeting the adult team before transfer were associated with increased participation and independence in formulating personal care plans [29, 32].

Adults with CP had a significantly higher rate of both GP visits and annual physicals compared to pre-transition young persons, but the total rate of physician visits was not significantly different between age groups [46]. Hospital utilization increased significantly among transitional age patients with CP in terms of number of annual discharges, inpatient days, and charges [33].

Comparatively, there was a significant decrease in both access and number of visits to specialists by young adults compared to pre-transition youth [37, 41, 46]. Specialists provided 28.4% of youth visits but only 18.8% of adult visits [46]. 34.7% of specialist referrals were unable to be made as such services did not exist for adult patients [34]. Adult physiotherapy and occupational therapy services were particularly under-provided [34]. Use of rehabilitation services decreased with age, independently of GMFCS status, while use of psychotropic and analgesic medication increased with age [32, 34, 36, 41, 43]. The highest areas of unmet needs in specialty care were for management of pain, bone or joint problems, and speech [42].

### Transition-associated social and functional outcomes

Young adults with CP significantly lagged non-CP peers in their development of housing, employment, and intimate relationships [32]. Use of formal respite services, support groups, and youth clubs was also relatively poor [37]. Social engagement decreased with age [43]. Living arrangements differed significantly among GMFCS levels, with 70% of participants with severe disability living with their parents [21]. Nonetheless, 55.9% of young adults with CP lived with their parents overall [21, 22]. Unmet needs in daily living healthcare and personal assistance were associated with increased severity of motor impairment (GMFCS) and attending non-specialist education [21, 42]. Unmet needs tended to increase over time but were not significantly related to whether the young person had transferred from child services [42]. One representative cohort of young adults with CP was 34.3% students, 19.6% employed, 35.2% participating in daily activities, and 8.8% unemployed, with 90% of employed participants having a GMFCS level of I–II [21].

### Qualitative interview key themes

Five studies [22, 23, 28, 35, 40] assessed attitudes of young adults with CP regarding their lived transition experiences. Shared themes included a lack of transition readiness and support, becoming a young adult and associated challenges, progression of physical disability, struggling to navigate the healthcare system, and feelings of isolation and abandonment.

Four studies [24, 31, 38, 43] assessed parents and caregiver attitudes towards their child’s transition from pediatric to adult healthcare services. Overarching themes included anxiety about service provisions, difficulty obtaining help and informational resources, poor communication and transparency from healthcare providers, the lack of coping skills, and a desire for community support. Parents also reported significantly lower satisfaction with current transition care processes than their children [38].

One study [27] included the provider perspective on transition challenges for young adults with CP. Themes included problems with accessibility, the suitability of primary care environments for caring for individuals with complex needs, gaps in seamless care, and limited time and funding when receiving or providing primary care services to young adults with CP.

### Outcomes of current transition programs

Four studies [26, 30, 39, 45] assessed current implementations of transitional care practice (Table 3). The top three perceived barriers to successful transition of care included limited adult providers willing to accept CP patients, concern about the level of care in the adult healthcare system, and the lack of financial resources [26]. One survey [26] of 11 clinics across the USA found that 55% of clinics had structured transition programs, but only one program successfully transitioned 100% of their patients to adult providers by age 22. Forty percent of the clinics had transitioned < 25% of their patients with CP to adult providers by age 22 [26]. Only one clinic had an absolute upper age limit for seeing patients, and 36% of clinics accepted new patients older than 21 [26]. None of the responding providers were “completely satisfied” with their transition process [26].

Significant gaps existed between the advertised services and the experiences of young people utilizing them [30]. Proposed beneficial features of transition services included a written transition plan, holistic life-skills training, and having a key worker, which only 30, 43, and 49% of young people agreed were available to them, respectively [30]. Agreement was better for appropriate parent involvement, age-banded clinic, promotion of health self-efficacy, and coordinated team at 77, 77, 80, and 69%, respectively [30]. Comparatively, a retrospective evaluation [39] of another transition clinic demonstrated that more than 2/3 of families reported receiving services across seven categories: support primary care, specialty care, school, legal, community inclusion, healthcare financing, and providing medical care. Key areas identified for improvement included coordination and communication between and within health services [45].

## Discussion

We present a systematic review of the state of transitional care for young persons with CP. To the best of our knowledge, this is the first systematic review on the topic. We emphasize three primary findings. First, the current state of transitional care for young adults with CP is inadequate, a perception shared by patients, caregivers, and providers. Second, key barriers to successful transition include poor communication between pediatric and adult provider teams, the lack of resources for coordinating and navigating care or for creating space for accommodating physical needs, and few providers for patients with CP or education on caring for adults with CP. Third, critical interventions to improve transitional care include early discussion and transition planning with pediatricians, community engagement and training, assigning a patient navigator or case worker to transition-age patients, and establishment of best practice guidelines to standardize existing transition programs.

### Current state of transitional care

Consistently, young adults with CP have expressed not feeling ready for the transition to adulthood [22–24, 27, 28, 31, 35, 40, 43, 44], as well as a desire for a comprehensive transition approach that prioritizes capacity building and personal empowerment [22–24, 28, 29, 31, 35]. However, the results of this review demonstrate that the current state of transitional care is insufficient to meet the complex needs of young adults with CP. While many transition-age persons with CP and their caregivers are actively seeking both primary and specialty care, there is poor access [25–27, 30–38, 40–43]. Medical providers with the expertise, office and financial resources, and capacity or willingness to see young adults with CP are limited in quantity and location [25–27, 30–38, 40–43]. Furthermore, such providers may be difficult to identify, have long waiting lists, or be financially inaccessible. As a result, these patients are frequently being seen in the emergency room instead of by trained specialists equipped to manage their complex care needs [33]. While hospital staff may be able to manage short-term, emergent care in these patients, long-term goals of care and management are rarely addressed in this setting [48].

The impact of opportunities for improvement in transitional care is further illustrated by the social and functional outcomes of study participants after aging out of the pediatric system. A lack of early intervention to promote social and career development leaves many young adults with CP unable to manage independent living, build careers, form intimate relationships, or engage socially [21, 22, 24, 32, 37, 40, 43]. These findings were magnified in those with more severe disabilities [21, 32]. However, current literature suggests that many adults with CP can live independently and have a high quality of life, especially if they have a strong support system and continuous specialty care [49]. As such, transitional care for young adults with CP should include means to address their social, emotional, and financial needs as they navigate adulthood.

Those few structured transition-programs that do exist are notable, and geographic location and financial resources restrict wider availability [26]. Many of these programs accept patients with a wide range of chronic illnesses, so management may be difficult to tailor to individuals’ disease-specific needs [50]. Most clinics in the USA are in major cities, affiliated with large, well-resourced, academic institutions, and thus primarily serve urban populations. These factors can create additional barriers to access, including physical distance or financial challenges for travel. Even those with access to these programs report low rates of satisfaction with the transition process and support services [26, 30, 45], a sentiment reflected by caregivers and providers alike [24, 26, 27, 30, 31, 39, 43]. There is a need for continued evaluation and reform in transitional healthcare.

### Barriers to successful transition

Many young adults with CP are lost to follow-up upon graduating from the pediatric healthcare system. The level of care coordination and close monitoring provided during childhood is often not continued or available to adults or older age groups, even in countries with universal healthcare coverage [51]. Many patients and their families may struggle to leave pediatric providers and multidisciplinary care teams with longitudinal relationships they have been with for their entire life and establish trust with a new provider or ecosystem. Release of records may be delayed or forgotten altogether as the time interval between providers grows [35]. As a result, transition planning tools that may have been constructed with the patient’s pediatric team are not optimally utilized. In addition, frameworks for transition of care exist, so the application of transition planning is not prescriptive. For instance, GotTransition.org outlines 6 domains, without a specific detailed way of making it happen. The implementation is thus variable based on many factors, including individuals, providers, and health system factors, which leaves the outcome somewhat to chance.

Young adults with CP struggle to navigate the complex terrain of the adult healthcare system, which assigns, functions, and delivers healthcare in significantly different ways than the pediatric system. Cognitive delay and worsening physical disability can create additional challenges for patients in the domains of self-advocacy, scheduling appointments, and coordinating their own care [51]. Aging out of “dependent” status on parent or caregiver insurance plans leaves many young adults with CP on public aid, requiring them to adapt their healthcare needs to newly limited provider coverage and benefits [51]. There is also generally greater support from ancillary services in pediatric care, such as social work, nurse coordinators, and resources for caregivers. However, similar resources are largely limited in the adult healthcare system, leading to increased demands on caregivers, stress, burnout, and difficulty managing their adolescent’s changing needs [24, 27, 43].

Providers also face obstacles in providing care, emphasizing lack of time, resources, financial reimbursement, and training [27, 52, 53]. Providers may consider themselves ill-equipped to manage young persons with CP due to limited education on treating spasticity, dystonia, and complex orthopedic issues in this population [53–55]. Additionally, persons with CP often require wheelchair-accessible entrances, exam rooms, exam equipment, and office personnel trained in proper patient handling techniques. These patients may also require longer appointments or have public insurance, which some providers may struggle to accommodate [27, 52, 53]. While the Americans with Disabilities Act (ADA) requires medical service providers in the USA to make their services accessible [56], similar statutes do not exist in every country or may be upheld to varying degrees [57].

### Opportunities for intervention

Adults with CP require the expertise of both pediatric and adult-trained providers who are familiar with the pediatric and adult manifestations of the condition. As such, collaboration between pediatric and adult providers is crucial to transition success [26, 33]. Pediatricians should begin the discussion and planning for transition to adult healthcare early in adolescence, so as to identify possible barriers to care early on [25]. Additionally, pediatric providers can actively seek out adult care providers in their communities with an interest in caring for patients with CP to start a dialogue focused on potential barriers to care and work towards building local networks of CP providers [26]. Such a network could assist in facilitating patients meeting their adult teams prior to transfer, which has been significantly associated with increased participation in organizing their own care [29]. Convening a national focus group of pediatric and adult care providers to discuss and publish optimal transition processes could also be valuable [26].

Adult-training programs should continue to implement teaching on adult manifestations of pediatric conditions to accurately reflect the needs of today’s patient population. Mandating transition training at both the student and resident level, either via direct incorporation into a national curriculum or creation of a specialty certification program, is needed [55]. Essential elements of such a curriculum would include patient-family involvement in teaching, addressing critical issues regarding financing, and discussion of medical decision making for adults with varying degrees of dependence on caregivers and illness progression [58, 59]. Community providers with experience transitioning young persons with CP should serve as instructors and faculty mentors across specialties [55]. Certification could also be accessible via the completion of online training modules [58]. As transitional care for this population requires a multidisciplinary healthcare team, students and residents should practice providing and coordinating care in multidisciplinary settings [54, 59]. Mentors should assist students and residents interested in advocacy projects to identify opportunities for greater involvement and funding. In doing so, we can ensure there are ample adult providers with the comfort and competence to treat young adults with CP [25, 54].

Furthermore, transition of care protocols should be usable across various clinics and geographic areas, and not merely successful in one clinic or hospital system [26]. Once best practice guidelines have been established, standardized transition clinics designed around the complex care needs of adolescents and young adults with CP can be established. A comprehensive clinic would ideally address the health, vocational, and daily living needs of young adults with CP, including counseling, therapy, legal, and financial support services [22]. The provision of a “navigator” for parents and adolescents during their transition to adult healthcare services could be immensely helpful in facilitating use of multiple services [22, 24]. This intervention has been effective in transitional care for young adults with other chronic diseases of childhood, resulting in decreased loss to follow-up and significant improvement in mean transition readiness scores, disease knowledge, and confidence in both disease and pain management [1, 60]. Further quantitative and qualitative research, including possible trials and implementation science collaborations, are needed to elucidate the most effective interventions and program structure.

Recommendations for transitional care practices, based on the findings of this review, are summarized in Table 4.

**Table 4:** Themes with cross-study consensus, associated subthemes, and recommendations for successful healthcare transition in young persons with cerebral palsy

**Global themes and subthemes**			
	**Overarching theme**	**Subthemes**	**Recommendations**
**Patients**	Transition readiness	Attachment to pediatric team Fear of abandonment Lack of external support Not knowing what happens next	Transition should be timed based on clinical milestones and emotional readiness, jointly determined by provider, patient and caregiver assessment, as opposed to age. Meeting the adult team before transfer, improved communication between pediatric and adult providers, written transition planning, and patient education on personal disease management may aid in transition readiness. Establishment of a comprehensive, standardized transition readiness assessment could also be useful
	Becoming a young adult	Progression of physical disability Stereotypes and prejudice Locating support for post-secondary education and employment Living independently—exciting but intimidating	Successful transition should be holistic with emphasis on functional, social, and emotional support, as well as teach financial and healthcare planning to empower young adults to be self-sufficient. As transition clinics and longitudinal programs for young adults with CP are developed, these elements should be incorporated into the care model
	Navigating the healthcare system	Encouraging independence but with limited guidance Locating experienced providers Understanding insurance benefits	Successful transition must account for the individual’s barriers to care, including access and availability of specialists, proximity to healthcare facilities, disparity in policy, insurance restrictions, stigma, and financial resources. Assigning a care coordinator or transition navigator to each patient could alleviate stress and confusion and lend to more successful transition
**Caregivers**	Isolation and abandonment	Identifying supportive resources Communication with providers Coping skills Community support	Assigning care coordinators or transition navigators to patients and their families, forming parent support groups as part of transition clinics or as online communities, and improved provider education on caring for young adults with CP can help alleviate caregiver stress and burnout
**Providers**	Accessibility	Care environment suitability Available time Funding	Expansion of public insurance benefits, healthcare policy reform, and increased private and public funding sources are needed to support the financial burden of providing complex care
	Training	Provider education Network for seamless care	Development of accessible educational resources, such as a free, online certification course, and incorporation of transitional care training into the teaching curriculum of residency programs is needed to expand the network of providers comfortable treating young adults with CP. Providers with this special certification are added to a searchable registry for patients seeking care

### Limitations

There are several limitations to this review. As only published full-text studies were included, results are at risk for publication bias. Studies not written or translated into English were excluded from this review, potentially resulting in missed findings on the presence and efficacy of transition programs in other countries. Included studies had an overall moderate risk of bias given that the majority were descriptive or qualitative in nature. None of the included studies were randomized controlled trials, which are necessary to provide the highest-quality evidence on successful transition measures and associated outcome data. These elements limit our ability to detect potential differences in race, gender, or institution. Some of the included studies excluded children with varying degrees of cognitive impairment and/or functional status. Additionally, there was extensive variety in the measures and tools used to assess transition readiness across studies. Data from low- or middle-income countries were lacking. While many high-income countries have begun to implement healthcare transition-type programs, a notable worldwide gap still exists due to limited data from resource-limited areas [61, 62]. Despite these limitations, strict PRISMA guidelines were followed to systematically assess and provide a comprehensive analysis of the published literature. Future studies should develop and evaluate contextually appropriate, comprehensive, transitional readiness tools and care models [63–66].

## Conclusions

Although people with CP may attain a high quality of life and a degree of independence, current transitional care paradigms are often insufficient. A flexible, individualized, transition period should be employed for each person with CP. Additionally, there is a need for evidence-based transition strategies, long-term care planning, and financial and educational resources.

### Supplementary Information

Below is the link to the electronic supplementary material.

Supplementary file1 (DOCX 12 KB)

## Data Availability

Data regarding the systematic review can be available upon request.
