# Supplementary material for: Gaps in transitional care to adulthood for patients with cerebral palsy: a systematic review
Source: Childs Nerv Syst. 2023 Aug 8;39(11):3083–101. doi: 10.1007/s00381-023-06080-2 (PMC10643351; doi:10.1007/s00381-023-06080-2)
Supplement: Supplementary file 1 — Supplementary file1 (DOCX 12 KB) [file 381_2023_6080_MOESM1_ESM.docx]

**Supplementary Table 1.** Search terms used for the three respective databases included in this systematic review.

| **Database** | **Search Query** |
| --- | --- |
| **PubMed** | (“cerebral palsy”[MeSH] OR “cerebral pals*”[tiab] OR “spastic displegia*”[tiab] OR “little’s disease*”[tiab])  AND  (“transition to adult care”[MeSH] OR “continuity of patient care”[MeSH] OR “transition*”[tiab] OR “continuit*”[tiab]) |
| **Embase** | (“cerebral pals*” OR “spastic displegia” OR “little* disease”):ti,ab,kw  AND  (“transition*” OR “continuit*”):ti,ab,kw |
| **Scopus** | TITLE-ABS-KEY(  ((“cerebral pals*” OR “spastic displegia” OR “little’s disease*”)  AND  (“transition*” OR “continuit*”)  ) |
